# Coding of Sexual Assault by Emergency Physicians: A Nationally Representative Study

**DOI:** 10.5811/westjem.2020.12.49045

**Published:** 2021-03-04

**Authors:** Vithya Murugan, Katherine J. Holzer, Michael G. Vaughn, Jason T. Carbone, Dylan B. Jackson, Cindy C. Bitter

**Affiliations:** * Saint Louis University, School of Social Work, St. Louis, Missouri; † Washington University School of Medicine, Division of Clinical and Translation Research, St. Louis, Missouri; ‡ Yonsei University, Graduate School of Social Welfare, Seoul, Republic of Korea; § Wayne State University, School of Social Work, Detroit, Michigan; ¶ Johns Hopkins University, Bloomberg School of Public Health, Baltimore, Maryland; || Saint Louis University School of Medicine, Division of Emergency Medicine, St. Louis, Missouri

## Abstract

**Introduction:**

Sexual assault is a public health problem that affects many Americans and has multiple long-lasting effects on victims. Medical evaluation after sexual assault frequently occurs in the emergency department, and documentation of the visit plays a significant role in decisions regarding prosecution and outcomes of legal cases against perpetrators. The American College of Emergency Physicians recommends coding such visits as sexual assault rather than adding modifiers such as “alleged.”

**Methods:**

This study reviews factors associated with coding of visits as sexual assault compared to suspected sexual assault using the 2016 Nationwide Emergency Department Sample.

**Results:**

Younger age, female gender, a larger number of procedure codes, urban hospital location, and lack of concurrent alcohol use are associated with coding for confirmed sexual assault.

**Conclusion:**

Implications of this coding are discussed.

## INTRODUCTION

Sexual assault and rape remain public health and medical crises in the United States. Empirically documented correlates of sexual victimization include young age, female gender, childhood history of maltreatment, and substance use/abuse.^1^ Approximately 20% of women and 2% of men experience rape at some point in their lives, accounting for an estimated 1.2 trillion dollars in direct medical costs and a total of 3.1 trillion dollars when lost productivity and other indirect costs are included (2014 US dollars).^2^,^3^ Despite the deleterious and long-lasting physical and mental health conditions associated with rape and sexual assault, most of these assaults are never reported.^4^–^6^ According to Kimerling, the under-reporting of sexual assault may be attributed to the “private, intimate nature of the assault and pervasive negative social consequences to disclosure.”^7^ While it is widely accepted that sexual assault and rape are under-reported, medical personnel, law enforcement, the legal system, and society are often skeptical when victims do come forward.

The current legal definition of rape stresses lack of consent and does not require the use of force,^8^ and the medical literature is clear that the presence of associated injuries is not required to prove the occurrence of sexual assault.^9^–^12^ The reported incidence of genital and other injuries associated with sexual assault is widely variable and depends on the methods used to detect injuries.^13^ However, victims are more likely to report the sexual assault and law enforcement is more likely to pursue investigation if there are associated physical injuries.^14^–^16^ Guidelines developed by the American College of Emergency Physicians recommend coding encounters as “sexual assault” rather than using modifiers such as “alleged” or “rule-out” sexual assault.^17^ Given the importance of medical documentation on future legal proceedings, we sought to determine factors that are associated with coding sexual assault vs alleged sexual assault.

## METHODS

This study employed data from the 2016 Nationwide Emergency Department Sample (NEDS) of the Healthcare Cost Utilization Project (HCUP) distributed by the US Department of Health and Human Services Agency for Healthcare Research and Quality (AHRQ).^18^–^20^ The NEDS includes data on approximately 33 million hospital-based ED visits from 953 hospitals approximating a 20% sample of US hospital-owned EDs. The sample is stratified by geographic region, trauma center designation, urban-rural location of the hospital, teaching hospitals, and hospital ownership. HCUP provides the hospital and discharge information necessary to calculate national estimates of ED visits, along with demographic information, reason for ED visit, and charge information. The analytic sample consists of individuals who were discharged from US EDs in 2016 with a diagnostic code of either suspected (n = 5948, weighted n = 26,421; 95% CI, 21,847–30,995) or confirmed sexual abuse (n = 5781, weighted n = 24,627; 95% CI, 21,254–28,000).

### Measures

#### Confirmed Sexual Abuse

Each record contained in the NEDS can include up to 30 *International Classification of Diseases, 10^th^ Revision, Clinical Modification* (ICD-10-CM) (World Health Organization, 1993) diagnostic codes, each representing a different diagnosis. A single dichotomous code was used to identify cases with a diagnosis of confirmed sexual abuse (ICD-10-CM code T74.21).

#### Suspected Sexual Abuse

A single dichotomous code was used to identify cases with a diagnosis of suspected sexual abuse (T76.21).

#### Patient Covariates

Patient characteristics included age in years (>25, 26–35, 36–50, and >50), gender, ZIP code median household income quartile, and insurance status (Medicare, Medicaid, private, and self-pay, no charge, or other). As a marker for more severe injury, we also included the number and types of procedures coded on the patient’s discharge record using the Common Procedural Technology (CPT) or Healthcare Common Procedure Coding System (HCPSC) collection of codes.

#### Alcohol Use and Abuse

Dichotomous variables (*yes*, *no*) were created for alcohol use and alcohol abuse diagnostic codes. Patients with the diagnostic code for alcohol use, unspecified (F10.9) were coded as having alcohol use. Patients were coded as having alcohol abuse if they had the F10.1 diagnosis code labeled alcohol abuse.

**Population Health Research Capsule**

What do we already know about this issue?

*Guidelines for the care of sexual assault survivors recommend coding visits as “sexual assault” rather than modifiers that imply uncertainty*.

What was the research question?

What factors are associated with coding of sexual assaults by emergency physicians?

What was the major finding of the study?

*Less than half of sexual assault visits are coded as “confirmed.” Demographic factors and alcohol abuse account for a small percentage of the variation*.

How does this improve population health?

*Sexual assault is underreported. Documentation that casts doubt on survivors may increase stigma, decrease engagement with follow up, and impede criminal justice proceedings*.

#### Hospital Characteristics

The hospital’s urban-rural designation, trauma level, and teaching status were included in the analyses. The urban-rural status of a hospital is based on the county of the hospital identified by the American Hospital Association (AHA). Hospitals in large metropolitan areas with at least one million residents and those in small metropolitan areas were considered urban. Hospitals in micropolitan and non-urban residual areas were classified as rural in the present analyses. The hospital trauma level designation was obtained from the Trauma Information Exchange Program database. For this study, trauma center Level I or Level II were grouped as trauma centers, and non-trauma centers and Level III were considered non-trauma. Teaching status is classified by the AHA Annual Survey of Hospitals as metropolitan non-teaching, metropolitan teaching, and non-metropolitan hospital. For the present study, we combined metropolitan non-teaching and non-metropolitan hospital into a non-teaching group.

### Analyses

We conducted bivariate analyses to identify the prevalence of a diagnosis of confirmed sexual abuse or suspected sexual abuse among ED patients as well as provide descriptive statistics for these individuals. Multivariate logistic regression was conducted to investigate sociodemographic and hospital-level differences between individuals with confirmed sexual abuse or suspected sexual abuse. The analyses were weighted to account for the NEDS complex sampling design using the *svyset* command and *svy* prefix in Stata 14.2 (StataCorp, College Station, TX).

## RESULTS

In 2016, there were approximately 26,421 adult discharges from EDs with a diagnostic code for suspected sexual abuse and 24,627 with confirmed sexual abuse. Table 1 presents estimates of the association between suspected sexual abuse and confirmed sexual abuse regarding key sociodemographic and hospital-level factors. With respect to age, those with confirmed sexual abuse were more likely to be younger than 25 compared to any other age group. With respect to gender, those with confirmed sexual abuse were significantly more likely to be female than male (odds ratio [OR], 1.18; 95% confidence interval [CI], 1.02–1.36); however, this difference is no longer significant when adjusting for sociodemographic and hospital factors. Individuals with confirmed sexual abuse are 28% less likely to be diagnosed with alcohol abuse than individuals with suspected abuse (adjusted OR [AOR], 0.72, 95% CI, 0.56–0.93); however, no significant difference was observed between the groups with regard to alcohol use. The number of CPT procedures appears to be slightly higher for individuals with confirmed sexual abuse. No differences were observed for ZIP code median household income quartile.

With regard to hospital-level characteristics, individuals with confirmed sexual abuse are significantly more likely to admit to an urban hospital compared to one in a rural area (AOR, 1.59; 95% CI, 1.15–2.18). Teaching status or trauma level of the hospital was not significantly associated with suspected vs confirmed sexual abuse.

## DISCUSSION

Using the NEDS database, we estimated there were 24,627 ED visits for confirmed sexual abuse and 26,421 visits for suspected sexual abuse in 2016, a total of 51,048 ED visits. In the same time period, there were 130,603 rapes reported to the Federal Bureau of Investigation.^21^ The most recent National Intimate Partner and Sexual Violence Survey estimated that 1,484,000 women are raped annually.^22^ In this context, we sought to determine factors that are associated with coding-confirmed sexual assault vs alleged sexual assault. We found that younger age, female gender, higher number of procedural services, urban hospital location, and lack of associated code for alcohol abuse were significantly associated with a code of confirmed sexual assault.

Regarding age, it is not necessarily surprising that individuals between the ages of 18–24 are more likely to receive a code of confirmed sexual abuse. Extant data suggest that most sexual assaults against females occur prior to the age of 25.^1^ Furthermore, the age group of 18–24 is referred to as “emerging adults” characterized by significant life transitions including college attendance and/or entry into the workforce in addition to increased experimentation and participation in unsafe behavior (ie, substance use).^23^–^25^ As a result, this age demographic is particularly vulnerable to both violence and substance use.^25^ In recent years, sexual assault on college campuses has garnered significant national attention with increased media coverage and programming and services.^26^,^27^ Provider willingness to code cases with younger victims as sexual assault rather than adding the modifier “alleged” may reflect awareness of and empathy for these vulnerabilities.

In this study, female victims were more likely to be coded as sexual assault than male victims, although this difference was not significant when adjusted for sociodemographic and hospital factors. This finding may be reflective of provider inexperience with sexual assault against males due to the small number of cases. Alternatively, it may be indicative of biases based on gender norms that stigmatize male victims. These biases may serve as barriers to male victims seeking care and perpetuate the myth that males are less likely to experience sexual assault.

Cases coded as confirmed sexual assault had slightly more CPT procedures than cases coded as alleged. However, supplemental analysis of the exact CPT codes used did not suggest more severe injuries in cases coded as sexual assault as billing level/medical complexity and procedures were similar between groups. Hospitals in urban areas were more likely to code for confirmed sexual abuse. Research suggests that rural areas have more geographic and economic barriers to seeking healthcare.^28^,^29^ Furthermore, a lack of Sexual Assault Nurse Examiner (SANE)-trained nurses in rural areas has been empirically documented and may explain the difference in confirmed vs alleged sexual assault coding for sexual abuse between urban and rural hospitals.^30^

Sexual assault victims with a concurrent code for alcohol abuse were less likely to be coded as confirmed. Alcohol intoxication may cause impairment in the ability to give active consent to participate in sexual activity. Additionally, alcohol intoxication may increase uncertainty regarding the events surrounding the assault. Nevertheless, alcohol intoxication does not negate reports of sexual assault. Moreover, alcohol intoxication may lead to delayed presentation to the ED after an assault, for fear of not being credible or facing negative consequences (ie, underage drinking). Delayed help-seeking may affect victims’ abilities to have crucial forensic evidence collected (ie, SANE exam).

This study suggests that slightly less than half of ED visits for evaluation after sexual assault are coded as “confirmed.” Younger age, female gender, and urban location of hospital were associated with higher rates of coding as confirmed but ORs were low, and it is important to remember that false allegations of rape occur in 2–10% of cases, a rate similar to false reports for other crimes.^31^,^32^ Given the importance of the medical documentation, it is crucial that the ED record reflect the events as reported by the victim, corroborated by physical exam findings when present. Only a small minority of victims present to the ED for evaluation after sexual assault, and a poor interaction with the healthcare system may adversely impact victims’ future mental health as well as success of legal proceedings.^33^,^34^

Similar to findings in Rudman et al’s work on coding of domestic violence, providers may be reluctant to code cases as confirmed sexual assault due to fears of stigmatizing the patient, unwillingness to commit to the diagnosis in face of uncertainty, or fear of medicolegal liability.^35^ Inadequate experience with sexual assault victims and the forensic exam during residency training may also contribute to uncertainty as to proper coding.^36^ This highlights the need for additional training regarding the importance of appropriate documentation and coding of sexual assaults. Directions for future research include qualitative studies of providers’ rationale for their documentation and coding practices, evaluation of training programs intended to improve documentation, and use of telehealth to expand access to SANE programs.

## LIMITATIONS

The study relies on the NEDS database with the inherent limitations of use of administrative databases for research purposes.^37^–^39^ NEDS relies on complete and accurate coding by participating institutions but coding may be incomplete. Rates of diagnostic testing and screening may vary across providers and/or institutions. For example, providers may differ in their use of screening, brief intervention, and referral to treatment for at-risk alcohol use, which could affect the likelihood of being coded as alcohol use vs alcohol abuse. Information about the availability of SANE nurses is not included in the NEDS database and it was not possible to correlate coding to assessment by a SANE nurse.

We identified correlates to coding confirmed sexual assault but such associations cannot determine causality. Additional correlates that may predict providers’ willingness to believe victims such as relationship of perpetrator to victim, provider gender, or confirmatory collateral history are not available from the database. Furthermore, data in the study are from 2016. Recent high-profile cases such as the Me Too movement and the outcry against lenient sentences imposed on perpetrators may have changed coding patterns.

## CONCLUSION

Our study underscores the necessity of accessible, accurate, and efficient ways to document sexual assault in EDs. Sexual assault is notoriously under-reported. Provider-level barriers coupled with the fast-paced nature of EDs may explain physicians’ reticence to code for sexual assault. Healthcare systems need to develop policies and practices that support ED providers in screening, treating, and providing appropriate referrals for sexual assault, with concerted efforts toward male victims and victims under the influence of alcohol. Rural EDs may ED identification of sexual assault has the potential to link victims to community services through referrals to counseling, victim advocates, and legal services – services that have been empirically documented to improve psychological health and increase social support.

## Figures and Tables

**Table:** Sociodemographic associations with suspected versus confirmed sexual abuse in United States emergency departments in 2016.

	Diagnostic code							
			
	Suspected sexual abuse (N = 5,948)		Confirmed sexual abuse (N = 5,718)		Unadjusted		Adjusted	

	Row %	95% CI	Row %	95% CI	OR	95% CI	OR	95% CI
Age								
<25	49.57	[44.56–54.59]	50.43	[45.41–55.44]	1.00	-	1.00	-
26–35	52.81	[47.75–57.81]	47.19	[42.19–52.25]	**0.88**	**[0.78–0.99]**	**0.86**	**[0.76–0.97]**
36–50	52.83	[48.13–57.49]	47.17	[42.51–51.87]	**0.88**	**[0.78–0.99]**	**0.86**	**[0.76–0.97]**
>50	59.37	[53.21–65.25]	40.63	[34.75–46.79]	**0.67**	**[0.56–0.81]**	**0.67**	**[0.56–0.81]**
Gender								
Male	55.50	[49.95–60.91]	44.5	[39.09–50.05]	1.00	-	1.00	-
Female	51.45	[46.72–56.15]	48.55	[43.85–53.28]	**1.18**	**[1.02–1.36]**	1.17	[1.00–1.37]
Insurance status								
Medicare	57.13	[52.22–61.90]	42.87	[38.10–47.78]	**0.74**	**[0.61–0.89]**	0.84	[0.68–1.04]
Medicaid	49.55	[45.87–53.23]	50.45	[46.77–54.13]	1.00	-	1.00	-
Private insurance	51.90	[47.59–56.18]	48.10	[43.82–52.41]	0.91	[0.78–1.06]	0.89	[0.75–1.05]
Other (self-pay, no charge, other)	52.60	[43.98–61.07]	47.40	[38.93–56.02]	0.89	[0.66–1.19]	0.88	[0.65–1.19]
Urban-rural								
Rural	60.67	[54.98–66.09]	39.33	[33.91–45.02]	1.00	-	1.00	-
Urban	49.95	[44.54–55.37]	50.05	[44.63–55.46]	**1.55**	**[1.12–2.13]**	**1.59**	**[1.15–2.18]**
Alcohol use								
No	51.69	[46.97–56.37]	48.31	[43.63–53.03]	1.00	-	1.00	-
Yes	60.94	[39.49–78.86]	39.06	[21.14–60.51]	0.69	[0.29–1.64]	0.66	[0.27–1.64]
Alcohol abuse								
No	51.45	[46.64–56.23]	48.55	[43.77–53.36]	1.00	-	1.00	-
Yes	59.61	[54.09–64.88]	40.39	[35.12–45.19]	**0.72**	**[0.57–0.91]**	**0.72**	**[0.56–0.93]**
Mean number of CPT procedures (SD)	5.62 (4.83)		6.05 (4.97)		1.02	[0.99–1.04]	**1.03**	**[1.01–1.05]**

Note: Odds ratios adjusted for age, gender, income, and location of hospital (urban or rural). Odds ratios and confidence intervals in bold are significant (P < 0.05).

*CI*, confidence interval; *OR*, odds ratio.
